# Type 1 Autoimmune Pancreatitis in a Young Male

**DOI:** 10.7759/cureus.7676

**Published:** 2020-04-15

**Authors:** Farooq Mohyud Din Chaudhary, Asim Tameez Ud Din, Khaleeq Siddiqui, Asma Tameez Ud Din, Sana Mohyud Din Chaudhary

**Affiliations:** 1 Gastroenterology, Mohyud Din Clinic, Multan, PAK; 2 Gastroenterology, Nishtar Medical University & Hospital, Multan, PAK; 3 Internal Medicine, Rawalpindi Medical University, Rawalpindi, PAK; 4 Internal Medicine, St. Joseph’s Medical Center, Stockton, USA; 5 Hematology, Combined Military Hospital Multan, Multan, PAK; 6 Internal Medicine, Combined Military Hospital Lahore Medical College & Institute of Dentistry, Lahore, PAK

**Keywords:** autoimmune pancreatitis, immunoglobulin type g4, recurrent pancreatitis

## Abstract

Autoimmune pancreatitis (AIP) is a rare entity leading to inflammation of the pancreas. It can be broadly categorized into two types. Type 1 AIP is more common and primarily presents with jaundice. Less commonly it can also progress to multiorgan involvement. Here we report a case of a 19-year-old male who presented to us with complaints of abdominal pain and vomiting. His laboratory investigations showed raised serum amylase and lipase. A contrast-enhanced CT revealed a diffuse enlargement of the pancreas with internal low-density foci. Due to the repeated episodes of pancreatitis, the patient's blood was tested for serum IgG4 (immunoglobulin type G4) which was markedly elevated pointing toward the diagnosis of AIP. Endoscopic ultrasound (EUS) showed a sausage-shaped pancreas with hyper- and hypoechoic strands. EUS-guided fine needle aspiration cytology of the lymph nodes performed in the celiac region showed a mixed population of lymphoid cells. Based on all the workup, our patient was diagnosed as type 1 AIP. He was managed with steroids and his condition progressively improved. This case is clinically significant because of the close resemblance of AIP with other pancreatic disorders like neoplasm. A timely diagnosis can prevent the unnecessary performance of invasive procedures in these patients.

## Introduction

Autoimmune pancreatitis (AIP) is a relatively uncommon form of inflammatory pancreatitis [1]. In a study conducted in Japan, the prevalence of AIP was found to be 0.82 per 100,000 [2]. It is differentiated in two types based upon the clinical and diagnostic work-up. Type 1 AIP typically presents in the adult population with common manifestation as jaundice. The serological immunoglobulin subclass 4 (IgG4) and lymphoplasmacytic sclerosing pancreatitis (LPSP) on histology are considered to be the hallmark features of type 1 AIP [3]. The resemblance of AIP with other pancreatic disorders like neoplasm poses a great challenge in diagnosing this condition [4]. It is also associated with multiple changes in the gallbladder and bile duct. A study conducted by Nishino et al. in the diagnosed cases of AIP showed gallbladder and bile duct wall thickening in 56% and 94%, respectively [5]. Here we present a case of a 19-year-old male who presented to us with complaints of abdominal pain and was diagnosed as a case of type 1 AIP after a detailed work-up. 

## Case presentation

A 19-year-old male patient presented to our hospital in July 2019, with complaints of abdominal pain and vomiting for the last 15 days. 

The patient had a history of recurrent abdominal pain for the last two years. Each episode was characteristic of severe central abdominal pain along with vomiting. He had multiple admissions as a result of these episodes. Detailed inquiry and checking of previous records revealed that these episodes were a result of recurrent attacks of acute pancreatitis. Each episode was characterized by markedly elevated levels of serum amylase and lipase, and imaging studies in the form of ultrasound and contrast-enhanced CT of the abdomen revealed a swollen pancreas and peripancreatic fluid collection. His imaging two years back revealed gallstones as well. Last year, he underwent endoscopic retrograde cholangiopancreaticography (ERCP) which showed gallstones and common bile duct (CBD) stones. Biliary stone removal and sphincterotomy were performed during the ERCP procedure. It was followed a few weeks later by laparoscopic cholecystectomy. He remained symptom-free for a few months. However, he again suffered from two further attacks of pancreatitis and underwent ERCP again which did not reveal any bile duct stones. 

Now, the patient presented to our department with complaints of severe central abdominal pain and vomiting for the last two weeks. The pain was of moderate intensity with radiation to the back. The pain was only relieved by taking narcotic painkillers. He also had multiple episodes of vomiting associated with food intake. There was no blood in his vomitus. The patient denied intake of alcohol, illicit drugs, or any type of alternative form of medicine. There was no history of trauma, insect bite, other procedures (apart from those mentioned above), headaches, altered level of consciousness, fever, cough, altered bowel habits, jaundice, skin rashes, or abdominal distension. He had lost around 10-kg weight in the last two years. Both of his parents had type 2 diabetes mellitus (DM). He did not smoke and belonged to a middle-class family. Due to these problems, he had left his studies about two years ago. 

On examination, the patient was clearly malnourished. He was in obvious distress. His vitals were as follows: blood pressure 130/80 mmHg, pulse 100 beats per minute, respiratory rate 22 breaths per minute, and temperature of 100°F. His abdomen was sunken with tenderness in the central part of his abdomen. His initial laboratory investigations are shown in Table 1. His serum calcium levels and lipid profile (including the triglyceride levels) were in the normal range. His contrast-enhanced CT of the abdomen showed diffusely enlarged pancreas with internal low-density foci, intrapancreatic calcification, and significant peripancreatic fat stranding. Based on his clinical presentation, laboratory investigations (raised amylase and lipase), and imaging, he was diagnosed as a case of acute pancreatitis. He was managed with intravenous fluids, painkillers, antiemetics, and proton pump inhibitors. Oral feeding was started as soon as the patient was able to tolerate it. Due to the repeated episodes of pancreatitis, the patient's blood was tested for serum IgG4 which was markedly elevated (Table 1), pointing toward the diagnosis of AIP.

**Table 1:** Laboratory Investigations ALP: alkaline phosphatase; ALT: alanine aminotransferase; anti-HCV: antibody to hepatitis C virus; AST: aspartate aminotransferase; BUN: blood urea nitrogen; HBsAg: surface antigen of hepatitis B virus; HCT, hematocrit; IgG4: immunoglobulin (type G4); INR: international normalized ratio; LDH: lactate dehydrogenase; MCHC: mean corpuscular hemoglobin concentration; MCV: mean corpuscular volume; PCV: packed cell volume; PT: prothrombin time; RBC: red blood cell; WBC: white blood cell.

Hematology Report		Blood Chemistry	
Hemoglobin	13.7 g/dL	Total bilirubin	0.3 mg/dL
RBC count	4.3 x 10^12^/L	ALT	25 U/L
HCT	42%	AST	25 U/L
MCV	86 fL	ALP	89 U/L
MCH	29 pg	LDH	143 U/L
MCHC	33 g/dL	Chemical pathology	
Platelets count	238 x 10^9^/L	Serum amylase	250 U/L (normal 23-85 U/L)
WBC	9.5 x 10^9^/L	Serum lipase	470 U/L (normal 0-160 U/L)
Neutrophils	74%	Renal function test	
Lymphocytes	14%	Serum creatinine	0.7 mg/dL
Monocytes	09%	Serum urea	21 mg/dL
Eosinophils	03%	BUN	10 mg/dL
Serum eectrolytes		Coagulation tests	
Sodium	137 mmol/L	PT	14 seconds
Potassium	3.8 mmol/L	INR	1.2
Chloride	106 mmol/L	Special test	
Bicarbonate	22 mmol/L	IgG4	3870 mg/L (normal 39-864 mg/L)
Viral serology			
HBsAg	Non-reactive		
Anti HCV	Non-reactive		

The patient was then referred to a specialized center for endoscopic ultrasound (EUS) and biopsy. EUS showed a sausage-shaped pancreas with hyper- and hypoechoic strands. The pancreatic duct was of normal size, 4 mm in the head and 1.7 mm in the body. Multiple peripancreatic lymph nodes in the celiac region and in the subhepatic region were seen. The largest one was 2.4 x 1.8 cm. CBD size was normal, and there was no evidence of CBD stone. There was no evidence of pancreatic divisum. Fine needle aspiration cytology (FNAC) of the lymph nodes was performed in the celiac region. Histopathology showed a mixed population of lymphoid cells. The pancreatic biopsy was not performed by the specialized center, and the reason they explained to us was that according to the diagnostic criteria, the patient had specific CT findings so only one more diagnostic test was required to confirm it and it was raised IgG4 levels. They performed FNAC of the lymph nodes to rule out any malignancy. Also due to a young age, the patient was not willing for more invasive tests. 

Based on all the work-up, our patient was diagnosed as type 1 AIP. He was started on steroids (tab prednisone 40 mg per day). His condition improved on steroids, and he was gradually tapered off of steroids in 12 to 16 weeks. 

## Discussion

AIP is one of the rare etiologies of pancreatitis. A number of diagnostic criteria have been identified to describe this entity. These include serologic markers like IgG4 level, rheumatoid factor, antinuclear antibodies, and a variety of imaging studies. In 2011, the "International Consensus Diagnostic Criteria" was introduced to design a comprehensive tool for evaluation and diagnosis of AIP. This consists of a combination of pancreatic parenchymal and ductal imaging, serum IgG4 levels, response to steroid therapy, and histology of pancreas. According to these criteria, pancreatic parenchymal imaging including CT or MRI is the foremost thing to do in a suspected case of AIP. If it manifests typical findings of type 1 AIP, which includes a diffuse enlargement of the pancreas, the presence of one other diagnostic evidence like raised serum IgG4 level or additional organ involvement points towards its diagnosis. Conversely, the presence of atypical findings and insufficient evidence mandates performing invasive tests like FNA, histology, and response to steroid therapy for further confirmation [6].

AIP can be subdivided into two types. Though there are some key differences with respect to epidemiology, clinical presentation, and serology, a definitive diagnosis can only be made on the basis of histology. Type 1 AIP typically presents in males with over 40 years of age and high serum levels of IgG4. The classic histological feature of type 1 AIP is LPSP [7]. Our patient was male with raised IgG4 levels and revealed a mixed population of lymphoid cells on EUS-guided FNAC of the lymph nodes in the celiac region. An interesting point was the young age of the patient which is atypical for type 1 AIP. 

Type 1 AIP can present with a myriad of clinical presentations but the most common is jaundice. Other symptoms include abdominal pain. Less commonly it can manifest as lesions in regions like a salivary, lacrimal gland, respiratory tract, and prostate [8]. Other extrapancreatic lesions mentioned in the literature include hydronephrosis, liver, and bile duct involvement causing sclerosing cholangitis and retroperitoneal fibrosis [9,10]. Our patient presented with fever, epigastric pain, and vomiting. A significant implication of AIP is secondary DM. A study conducted in Japan showed a high proportion of micro (retinopathy, nephropathy) and macrovascular complications in patients who had DM prior to the diagnosis of AIP [11]. 

Steroids are the mainstay treatment of AIP especially type 1. Kamisawa et al. demonstrated a significantly higher rate of response in patients treated with steroids as compared to the other group (98% vs 74%). After achieving remission, it is recommended to continue maintenance therapy for at least three to six months. The relapse rate in the patients, initially managed with steroids, was found to be 24% [12]. Other treatment modalities such as biliary stenting and immunomodulators can be used in patients not responding to steroid therapy. The management of associated diabetes and other organ involvement is also recommended in AIP [13]. Our patient showed improvement on the initiation of steroids. 

## Conclusions

AIP is a challenging diagnosis and can manifest with variable presentations. We present a case of a young male who presented to us with complaints of recurrent attacks of acute pancreatitis. He was diagnosed as a case of type 1 AIP based on the marked elevation of IgG4 level, the typical sausage-shaped appearance on EUS, and the presence of lymphoid cells on FNAC of the lymph nodes in the celiac region. He was started on steroids and his condition progressively improved.
